# Supraglottic Botulinum Toxin Improves Symptoms in Patients with Laryngeal Sensory Dysfunction Manifesting as Abnormal Throat Sensation and/or Chronic Refractory Cough

**DOI:** 10.3390/jcm10235486

**Published:** 2021-11-23

**Authors:** Daniel Novakovic, Meet Sheth, Thomas Stewart, Katrina Sandham, Catherine Madill, Antonia Chacon, Duy Duong Nguyen

**Affiliations:** 1 Voice Research Laboratory, Discipline of Speech Pathology, Faculty of Medicine and Health, The University of Sydney, Sydney, NSW 2006, Australia; meet.c.sheth@gmail.com (M.S.); thomas.e.stewart@sydney.edu.au (T.S.); cate.madill@sydney.edu.au (C.M.); antonia.chacon@sydney.edu.au (A.C.); duong.nguyen@sydney.edu.au (D.D.N.); 2 The Canterbury Hospital, Campsie, NSW 2194, Australia; 3 Sydney Voice and Swallowing, St. Leonards, NSW 2065, Australia; kate.sandham@gmail.com; 4 Department of Otolaryngology, Christian Medical College, Vellore 632004, India; 5 National Hospital of Otorhinolaryngology, Hanoi 11519, Vietnam

**Keywords:** laryngeal sensory dysfunction, chronic refractory cough, botulinum toxin, larynx, laryngeal hypersensitivity, cough hypersensitivity syndrome, globus pharyngeus, laryngopharyngeal reflux, neuropathic cough, throat irritation

## Abstract

Laryngeal sensory dysfunction (LSD) encompasses disorders of the vagal sensory pathways. Common manifestations include chronic refractory cough (CRC) and abnormal throat sensation (ATS). This study examined clinical characteristics and treatment outcomes of LSD using a novel approach of laryngeal supraglottic Onabotulinum toxin Type A injection (BTX). This was a retrospective review of clinical data and treatment outcomes of supraglottic BTX in patients with LSD. Between November 2019 and May 2021, 14 patients underwent 25 injection cycles of supraglottic BTX for treatment of symptoms related to LSD, including ATS and CRC. Primary outcome measures included the Newcastle Laryngeal Hypersensitivity Questionnaire (LHQ), Cough Severity Index (CSI), Reflux Symptom Index (RSI), and Voice Handicap Index-10 (VHI-10) at baseline and within three months of treatment. Pre- and post-treatment data were compared using a linear mixed model. After supraglottic BTX, LHQ scores improved by 2.6. RSI and CSI improved by 8.0 and 5.0, respectively. VHI-10 did not change as a result of treatment. Short-term response to SLN block was significantly associated with longer term response to BTX treatment. These findings suggest that LSD presents clinically as ATS and CRC along with other upper airway symptoms. Supraglottic BTX injection is a safe and effective technique in the treatment of symptoms of LSD.

## 1. Introduction

The larynx is innervated by branches of the vagus nerve with complex coordination of afferent (sensory) and efferent (motor) pathways in the brainstem required for optimal physiological functioning [1,2]. Neurological dysfunction can occur secondary to central or peripheral pathology affecting the vagal pathways. Depending upon the level and nature of injury, vagal dysfunction can have either or both sensory and motor effects manifesting within and outside the larynx. Motor manifestations of vagal dysfunction involving the larynx can be broadly classified into hypofunctional (e.g., vocal fold paralysis or paresis) or hyperfunctional (e.g., inducible laryngeal obstruction) with laryngeal movement disorders affecting higher centers. Sensory manifestations of vagal dysfunction are less well understood but can present independently or in conjunction with apparent motor effects.

Laryngeal sensory dysfunction (LSD) represents disorders of laryngeal afferent sensory pathways presenting with abnormal laryngeal sensation. Several phenotypes related to hyperfunctional vagal sensation have been described manifesting in the larynx sharing similar features [3]. These include chronic refractory cough (CRC) [4], various forms of inducible laryngeal obstruction including recurrent laryngospasm, paradoxical vocal fold movement [5,6] and irritable larynx syndrome [7], globus pharyngeus [3] and laryngeal sensory neuropathy [8] with various proposed etiologies. We prefer to use the umbrella term laryngeal sensory dysfunction [3] which recognizes the role of abnormal laryngeal afferent sensory pathways in these conditions which may be affected at one or more levels (peripheral receptors, afferent vagal fibers, central pathways), and which present with abnormal/altered laryngeal sensation, without attribution to a specific underlying pathological process or cause. Accurate evaluation of laryngeal dysfunction and hypersensitivity would allow for accurate diagnosis and effective treatment [9]. 

Laryngeal hypersensitivity has been best described in the context of CRC [10], which is defined as a cough persisting beyond 8 weeks despite guideline-based treatment. Other terms for CRC include neurogenic cough, idiopathic cough, psychogenic cough, habitual cough and (laryngeal) cough hypersensitivity syndrome [11]. Increased sensitivity of the afferent limb of the cough reflex has been demonstrated in CRC [4], with those affected exhibiting a lower cough threshold in the capsaicin challenge test [12,13]. Furthermore, Vertigan and Gibson observed abnormal laryngeal sensation (laryngeal paresthesia) in 94% of patients with CRC [4], consistent with a sensory neuropathic disorder.

The concept of sensory neuropathy causing laryngeal symptoms was first proposed by Morrison et al. using the term irritable larynx syndrome [7]. They described laryngospasm, dysphonia, globus pharyngeus, pain and/or chronic cough as potential symptoms arising from a hyperexcitable state of the laryngeal neuronal sensory network. Laryngeal sensory neuropathy has also been described in the context of hypofunctional laryngeal sensation associated with a high risk of dysphagia in head and neck cancer patients [14]. Neuropathy represents a disturbance of function or pathological change in one or more nerves which can change the normal sensitivity or thresholds of afferent nerves causing neuropathic symptoms. Neuropathic pain is characterized by the clinical features of paresthesia, hyperalgesia and allodynia [15] with equivalent laryngeal features manifesting as abnormal throat sensation, hypertussia and allotussia (Table 1) [4].

Laryngeal sensory receptors project centrally towards the nucleus tractus solitarius, primarily via the internal branch of the superior laryngeal nerve (iSLN), where activation can lead to a variety of reflexive responses including cough, swallow and laryngospasm [16]. The laryngeal adductor reflex (LAR) is one such robust and well-studied response which causes bilateral involuntary airway protective closure in response to supraglottic stimuli [17]. Topographic mapping of sensory receptors related to the LAR has recently been achieved. The highest density of LAR sensory receptors and afferent nerve fibers are found in the posterior supraglottis followed by the false vocal folds and epiglottic tip with no LAR activation with stimulation of the membranous vocal folds [18].

Peripheral and central sensitization are features of neuropathy. Peripheral sensitization describes both nociceptive and non-nociceptive sensory afferents becoming sensitized [15,19], with a lowered threshold for signaling, and/or an increase in the magnitude of responsiveness at the peripheral ends of sensory nerve fibers. A wide range of signaling molecules are involved in mediating peripheral sensitization including such neuropeptides as calcitonin gene-related peptide (CGRP) and substance P (SP). Laryngeal sensory dysfunction may occur at the periphery when laryngeal sensory receptors and nociceptive fibers become dysfunctional and undergo peripheral sensitization. The prolonged process of peripheral sensitization can lead to sensitization of the central sensory pathways, where potentiation by neurotransmitter signaling results in a net increase in neuronal spinal output [15,19].

### 1.1. Etiology of LSD/Mechanisms of Injury

The etiologies of LSD are yet to be fully elucidated, although numerous causes have been proposed in the literature. Morrison et al. [7] suggested viral infection, emotional distress, chronic reflux and habitual muscle misuse as potential contributing factors amongst other more common organic causes of nerve injuries [7].

Amin and Koufman [8] reported cases with laryngeal electromyographic evidence of lesions to both superior and recurrent laryngeal nerves. They maintained that damage to the vagal nerves was linked to a preceding viral upper respiratory tract infection as a one-off phenomenon rather than an ongoing/progressive degeneration or regeneration process [8]. Rees, Henderson and Belafsky [20] proposed Post-Viral Vagal Neuropathy as a clinical entity resulting from upper respiratory tract infection presenting with chronic cough, excessive throat clearing, dysphonia, and vocal fatigue with laryngoscopic signs of laryngeal motor weakness.

Honey et al. proposed neurovascular compression of the vagus nerve rootlets identified on magnetic resonance imaging [21] as a potential cause of vagal dysfunction presenting in the larynx with sensory symptoms of abnormal throat sensations [22] associated with motor symptoms of laryngospasm/choking, neurogenic cough or intermittent stridor [23].

Altman et al. suggested various factors (including topical airway infectious agents, inflammatory cytokines, viscosity of the airway mucus, gene regulation producing altered mucus in disease, the temperature and pH of the airway surface) may act synchronously to sensitize the larynx [24]. They activate and upregulate multiple upper airway receptors, including TRPV1 (transient receptor potential vanilloid 1, stimulated by acids, protons, and capsaicin). There is evidence that sensitization of the TRPV1 channel underlies hypersensitivity in neuropathic pain [25].

### 1.2. Assessment and Diagnosis of LSD

To date, no diagnostic criteria have been established for LSD. Consequently, the assessment of abnormal laryngeal sensation is based largely on patient history, clinical evaluation, appropriate questionnaires/patient-reported outcome measures (PROMs) and laryngeal investigations [9], along with limited response to treatment of other conditions which can present with similar symptomatology.

Several PROMs can be used for assessment of LSD (see methods). These questionnaires provide easily obtainable subjective baseline data which can then be used to monitor patient progress and treatment outcomes [26].

### 1.3. Superior Laryngeal Nerve Block

Local anesthetics are used extensively during endotracheal intubation and other procedures requiring upper airway manipulation to suppress normal physiological responses including cough and laryngospasm. Topical lidocaine (lignocaine) applied to the larynx has been shown to suppress laryngeal reflexes activated by mechanoreceptor and chemoceptor stimulation [27]. Superior laryngeal nerve block is another way to suppress these reflexes whereby the supraglottic larynx can be anesthetized in an awake patient by delivering local anesthesia around the internal branch of the superior laryngeal nerve at the thyro-hyoid membrane as it enters the larynx [28]. Lidocaine blockade of the SLN has been shown to temporarily relieve symptoms of laryngospasm due to known SLN injuries [29]. This opens the potential therapeutic pathway of modulating laryngeal sensation to treat conditions such as chronic refractory cough where LSD is a contributing factor. The temporary duration of this proposed modality as well as ease of administration makes this an excellent initial test to potentially predict response to treatments which can modulate sensation in the distribution of the SLN.

### 1.4. Treatment of LSD

Treatment of potential coexisting medical conditions that can present with similar symptoms is crucial in the management of LSD. A limited response will help support the diagnosis, but it is also important to control pathologies which can alter laryngeal sensitivity (including LPR, OSAS and chronic inflammation). Furthermore, any pathological process which can stimulate or irritate the laryngeal mucosa can act as a trigger of hypersensitized laryngeal sensory pathways and reflexes and reducing this sensory input can help with control of symptoms.

Centrally acting neuromodulators including amitriptyline, gabapentin, pregabalin and tramadol have some effectiveness in reducing symptoms linked to vagal neuropathy and have acceptance in the treatment of CRC [30,31]. There is evidence that gabapentin, which is effective mostly in pain due to nerve damage in postherpetic neuralgia and peripheral diabetic neuropathy [32], is also effective in treatment of odynophonia [8], neck pain [8], chronic cough and laryngospasm due to suspected sensory neuropathy of the SLN [33]. 

Behavioral treatment provided by a speech language pathologist (SLP) or physiotherapist has been found effective in management of CRC by reducing cough frequency [34] and cough reflex sensitivity [35]. Treatment typically includes some or all of the four elements described in the John Hunter Hospital Chronic Refractory Cough (JHCRC) Program: patient education regarding nature of cough, exercises to improve voluntary control over cough and/or suppression of the cough, reduction of behaviors that cause laryngeal irritation and psycho-educational counselling [36]. Improving voluntary control over one’s cough and reducing the sources of irritation that trigger coughing are complementary approaches that are of equal importance in alleviating this behavior [37]. The treating clinician must emphasize the commitment required for behavioral change to occur and provide additional supports as necessary to facilitate the patient’s independent management and control over their presenting symptoms. 

### 1.5. Botulinum Toxin in the Larynx, and Its Potential Role as a Sensory Neural Modulator

Onabotulinum toxin Type A (BTX) is a proteolytic enzyme that cleaves neuronal SNARE proteins which play a crucial role in the mediation of neurotransmitter release. The primary studied effect of BTX is in motor nerves, where neuromuscular conduction is inhibited by the toxin, resulting in a localized but reversible chemical denervation of the associated muscle fibers. 

The putative mechanism by which BTX may modulate laryngeal sensation can be best understood in the context of chronic refractory cough (CRC) and its correlation with neuropathic pain [30]. The therapeutic effects of BTX in CRC are thought to be due to its effects on sensory transmission and peripheral sensitization. Transient receptor potential (TRP) channels are a group of ion channels present on the plasma membrane of multiple mammalian cell types. In airway physiology, they play an important protective role in pathways inducing inflammation, mucus secretion, airway constriction, and reflexes such as cough and sneezing [38]. The reduced cough threshold in CRC is associated with increased expression of TRPV1 receptors on airway nerves [39,40]. Changes in these, and associated channels, along with the development of sensitization is the understood mechanism by which a chronic cough develops into a hypersensitivity syndrome [41].

In addition to motor effects, BTX also inhibits neurotransmitter release in sensory neurons, likely through the reduction in expression of neuropeptide transmitters, such as SP and CGRP. TRPA1 and TRPV1 [42] are associated with CGRP-dependent pathways. Administration of BTX has been demonstrated to disrupt the transfer of TRP receptors to synaptic membranes [43,44]. Studies have previously demonstrated that BTX reduces pain and neurogenic inflammation caused by capsaicin, which is the antagonist of TRPV1 receptors [45]. As such, BTX sensory mechanism is at least partially via its effect on TRPV1 expression, with this modulation likely also interrupting the process of peripheral sensitization [46]. BTX is also thought to affect central sensitization; however, this remains controversial [46,47]. The interruption of these sensitivity pathways by peripheral administration of BTX is a potential way to modulate the symptoms experienced under the umbrella term laryngeal sensory dysfunction.

BTX was first used in the larynx by Blitzer in 1984 as a treatment for adductor spasmodic dysphonia [48] (a focal laryngeal dystonia). It has since become the gold standard for this condition. Injections are usually targeted to the involved intrinsic laryngeal adductor muscles to weaken them and prevent inappropriate contractions causing disruption of normal speech.

Several studies have reported the use of BTX targeted to the laryngeal adductor musculature for the treatment of chronic refractory cough [49,50,51,52]. Delivery of BTX into the supraglottic region is a more recent concept and was initially described by Young and Blitzer in 2007 as an adjunct treatment for patients with adductor spasmodic dysphonia who exhibited sphincteric closure of the supraglottic larynx during phonation [53]. In 2016, Simpson reported supraglottic BTX as an alternative primary treatment for adductor spasmodic dysphonia [54], showing improved voice outcomes with a favorable side effect profile compared with glottic BTX. To date, no study has examined the sensory effects of laryngeal BTX when delivered into the supraglottis rather than into the intrinsic laryngeal musculature.

### 1.6. Current Study Aims

The present study investigated a novel treatment of supraglottic BTX for LSD. The aims of the study were to: (1) describe the clinical characteristics of LSD in a cohort of patients referred for CRC and abnormal throat sensation (ATS); (2) describe a new treatment of supraglottic laryngeal botulinum toxin in the symptomatic management of laryngeal sensory dysfunction; (3) evaluate the efficacy of using botulinum toxin A in treatment of a pilot group of patients presenting with different phenotypes associated with laryngeal sensory dysfunction including CRC and ATS. We hypothesized that CRC and ATS can be manifestations of LSD and that treatment aimed at LSD would have therapeutic effects quantifiable using patient reported outcome measures of cough and throat sensation. 

## 2. Materials and Methods

### 2.1. Study Design

This was a retrospective data review of an existing private specialized laryngology clinic database. The study was approved by the Human Research Ethics Committee of The University of Sydney (protocol number 2021/025).

### 2.2. Participants 

A database search was implemented to identify all patients who underwent supraglottic BTX injections as part of treatment for clinical presentations associated with LSD. 

Inclusion criteria were: (1) a history of sensory laryngeal symptoms (manifesting as CRC or ATS) for greater than 12 consecutive weeks despite assessment and treatment of potential/coexisting lower respiratory, sinonasal and laryngopharyngeal reflux pathology; (2) a Newcastle Laryngeal Hypersensitivity Questionnaire (LHQ) score of 17.1 or below [55].

Most patients had previously been offered neuromodulator medication and had either ceased this treatment due to poor response or negative side effects or remained on neuromodulators with partial symptom control whilst undergoing a trial of salvage laryngeal botulinum toxin treatment. All patients had been referred to a speech pathologist for behavioral treatment of their symptoms. Thirteen of the fourteen had seen a speech pathologist prior to BTX treatment. Speech pathology data was unavailable for one patient.

Fourteen patients were identified during the study period who underwent supraglottic BTX treatment for LSD, including six females and eight males. Mean age of patients was 54.9 years (standard deviation, SD = 12.5, range = 32–76).

**Figure 1:**
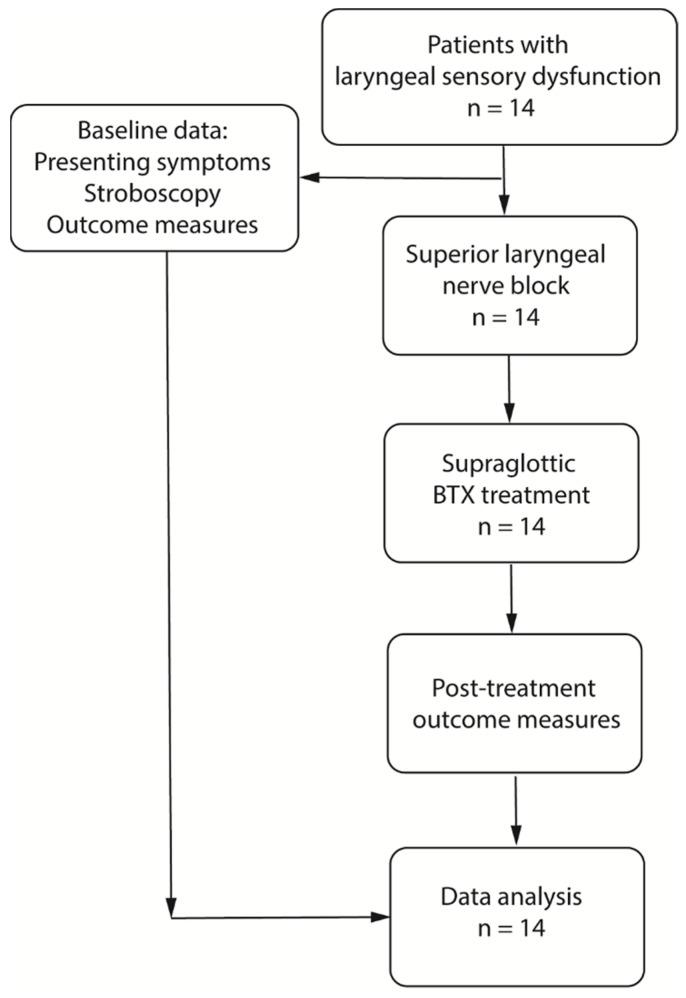
Flowchart of study protocols.

Figure 1 shows diagram of study protocols. Table 2 presents information regarding demographics, onset, respiratory pathology, and neural modulator treatment for all patients.

### 2.3. Intervention: Supraglottic BTX Injection

Patients presenting with LSD who had persistent symptoms despite medical and behavioral (speech pathology) management underwent trial superior laryngeal nerve (SLN) block in the clinic. Immediate response to SLN block was measured using a 10-point Likert scale questionnaire based upon the patient’s specific presenting symptoms which was developed using the Newcastle Laryngeal Hypersensitivity Questionnaire (LHQ) [55]. Immediate response was measured 20 min after SLN block and an improvement of their primary symptom by three or more points compared with baseline was considered a positive response. In the case of no response at 20 min, contralateral SLN block was offered, and response was assessed after a further 20 min. Patients who had symptomatic but short-term (<2 weeks) improvement after SLN block were offered subsequent botulinum toxin Type A (Botox™, Allergan, Irvine, CA, USA). Some patients who did not respond to SLN block elected to undergo a trial of supraglottic BTX treatment as salvage therapy after failed medical management including a trial of neuromodulator therapy.

**Figure 2:**
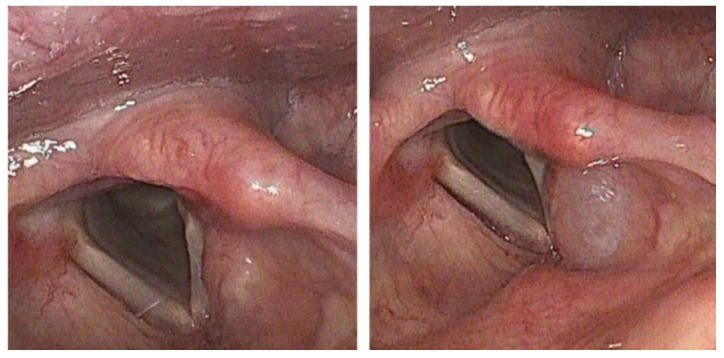
Endoscopic image of larynx before (**left**) and immediately after (**right**) supraglottic BTX injection showing visible submucosal bleb at injection site.

BTX was usually given in an office-based outpatient setting. (In one patient with extreme hypersensitivity to flexible laryngoscopy, the BTX injection was given trans-orally during microlaryngoscopy under general anesthetic). Patients were seated semi-reclined with the head extended. Decongestant with local anesthesia was administered topically to the nasal cavity (5% lidocaine + phenylephrine) prior to the procedure. Bilateral SLN blocks were performed using 2% lidocaine, 0.5 cc on each side for the purpose of anesthesia during the procedure. BTX injection was performed using a 1 cc syringe coupled to a 23 or 25 G needle which was introduced into the larynx via a trans thyro-hyoid approach with the needle directed inferiorly, posteriorly and slightly laterally toward the targeted supraglottic region of the false vocal fold and posterosuperior larynx—where sensory receptor density is thought to be highest [18]. Flexible transnasal videolaryngoscopy was used to help guide the injection into the desired region and confirm placement. The injectate was delivered whilst keeping the needle in a submucosal plane without breaching the laryngeal airway and correct placement was confirmed via the presence of a visible bleb at the injection site (Figure 2). The BTX concentration was kept constant at 2.5 U per 0.1 cc of injectate with dosage adjusted by varying volume of injectate.

Nineteen treatments were given unilaterally and six bilaterally (2 synchronous, 4 staged). Mean dose for each supraglottic injection was 7.74 U (SD = 1.75 U). Mean time of post-treatment assessment was 7.1 weeks (SD = 3.2 weeks). The decision on which side to treat with BTX and whether to treat unilaterally or bilaterally was made based on a combination of the following factors: (i) the patient’s self-perceived unilaterality of symptoms, (ii) laryngoscopic findings of motor asymmetry, particularly that of vertical height mismatch, with (iii) immediate response to SLN block on that side.

### 2.4. Data Extraction

One otolaryngologist and one registered nurse who were blind to the aims of the study performed data extraction from clinical records. The data described in the following subsection were collected during this review.

#### 2.4.1. Demographic Characteristics and History

Demographic characteristics (age, gender). Smoking history. Symptom duration and potential preceding factors. Past investigation/treatment of significant co-morbidities including gastro-esophageal or laryngopharyngeal reflux, lower respiratory tract pathology, sinonasal conditions and obstructive sleep apnea. Current/past medications including ACE inhibitors and neuromodulators.

#### 2.4.2. Videostrobolaryngoscopy Findings at Baseline

Videostrobolaryngoscopy is the gold standard clinical assessment for evaluating laryngeal structure and dynamic function [56]. All patients underwent neurolaryngological examination via trans nasal videostroboslaryngoscopy at baseline using a standardized clinical voice assessment protocol designed to identify potential features of laryngeal motor dysfunction [57]. Findings of vocal fold motion anomalies, glottic insufficiency and mucosal wave anomalies are the most reliable signs for the diagnosis of vocal fold paresis [56], a laryngeal motor impairment which may coexist with sensory dysfunction in some LSD patients where both efferent and afferent functions of the laryngeal nerve/s are affected.

All strobolaryngoscopy exams were extracted and blindly rated by two otolaryngologists using a tool developed in Bridge2practice, an online education and research platform developed for health and medical learning and practice of allied health professionals and students [58]. The following parameters were assessed: (1) vocal fold movement; (2) mucosal wave; (3) laryngeal muscle tension patterns.

Videos of eight strobolaryngoscopy exams were repeated, randomized and re-rated to evaluate intra-rater reliability. Ratings from the two blinded assessors were compared to calculate inter-rater reliability for stroboscopic parameters that are subject to low reliability of ratings such as vertical focal fold plane and phase symmetry [59]. Table 3 shows excellent intra-rater reliability and Table 4 shows good inter-rater reliability for key parameters.

#### 2.4.3. Outcome Measures

Several patient-reported outcome measures (PROMs) were used to evaluate laryngeal symptoms and were administered to all patients prior to BTX treatment and within 3 months of treatment. Where bilateral staged treatment was given, outcomes were measured after the second treatment.

(a) Newcastle Laryngeal Hypersensitivity Questionnaire (LHQ) [55].

The Newcastle Laryngeal Hypersensitivity Questionnaire (LHQ) scores 14 items across three specific domains: obstruction, pain/thermal and irritation, providing a robust measure of laryngeal sensory disturbance. This tool has proved useful in discriminating patients with laryngeal hypersensitivity from healthy people and in measuring changes in symptoms of laryngeal hypersensitivity following speech pathology treatment [55]. A normal score is considered to be 17.1 or above [55]. The clinically minimal important difference for this questionnaire is 1.7 [55].

(b) Cough Severity Index (CSI)

CRC is the context in which LSD has been most associated. The CSI [60] is a validated PROM commonly utilized in evaluating patients with CRC resulting from the upper airway and is proven to be sensitive in detecting treatment outcome [61,62]. A score of 3 or more is considered abnormal [60].

(c) Reflux Symptom Index (RSI)

The Reflux Symptom Index (RSI) is a validated PROM initially developed to measure symptom severity for laryngopharyngeal reflux (LPR) [63]. An RSI score >13 is considered abnormal [63]. Although not specific for LPR [64] it serves as a useful and commonly used marker of throat irritation with which it has been correlated [65] and a marker of symptomatic response to treatment [66].

(d) Voice Handicap Index 10 (VHI-10)

The Voice Handicap Index 10 is a validated PROM to assess patients’ perception of their voice function [67]. This tool was used in the present study given that patients with LSD and CRC frequently present with voice problems, e.g., muscle tension dysphonia [3]. It also allowed assessment of the frequency and severity of potential voice change which is a recognized potential side effect of laryngeal BTX treatment [68]. A score of greater than 11 is considered abnormal [69] with 6 considered as the minimal important difference [70].

### 2.5. Statistical Analyses

Data were managed in Microsoft Excel 365 [71] and analyzed using IBM SPSS Statistics v.24.0 [72] and Prism v8.1.2 [73] for Windows. Descriptive statistics were used to describe the cohort’s characteristics. Prior to analyses, normal distribution of the data was examined using Kolmogorov–Smirnov tests [74]. For continuous variables, mean, standard deviation (SD) and 95% confidence interval (normal distribution) or median and quartiles (non-normal distribution) were used. For categorical data, frequencies and percentages were used. Changes in outcome measures over the treatment period were analyzed using a linear mixed model with patients as random effects and time points (i.e., baseline and post-BTX injection) and gender as fixed effects. Interaction between ’time’ (treatment) and the fixed factors was also calculated to determine the impact of included factors on treatment outcome. Association between categorical variables was examined using Chi-square test (χ^2^). A significance level of two-tailed p of 0.05 was used. Where there were multiple calculations, Sidak-adjustment was applied to the *p* value. Effect sizes were calculated using Cohen’s d (small = 0.2; medium = 0.5; large = 0.8) [75].

## 3. Results

### 3.1. Characteristics of LSD

Table 5 presents primary presenting symptoms and secondary symptoms for all included patients. Primary symptoms were abnormal throat sensation (ATS) (12/14), followed by chronic cough (12/14) with a mean (SD) duration of 81 (110) months (min = 1; max = 360). Other symptoms included dysphonia (5/14), choking sensation (5/14), laryngeal dyspnea (5/14) and dysphagia (2/14).

Table 6 lists the results of PROMs at baseline and normative cut-off values from the literature. This table showed that the score values for these scales were well within the pathological ranges.

Table 7 shows findings for the relevant strobolaryngoscopy parameters. The predominant clinical feature on strobolaryngoscopy observed in 10/14 participants was vertical mismatch of the vocal folds, followed by some form of lateral or medial constriction of the supraglottic structures during phonation. Abduction lag and unilateral false vocal fold hyperfunction were observed in 6/14 participants and 5/14 participants were observed to have one vocal fold shorter than the other. Phase asymmetry and reduced mucosal wave amplitude were not features found in this population.

### 3.2. Effects of Botox Injection on Outcome Measures

#### 3.2.1. LHQ Score

**Figure 3:**
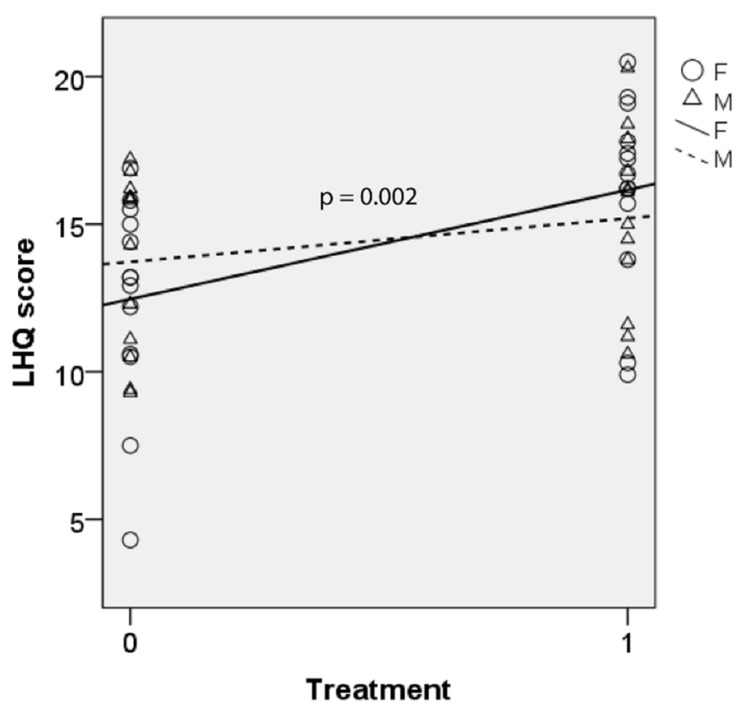
LHQ scores before and after BTX therapy with linear trend lines for male (M) and female (F). Higher score means better outcome. 0 = baseline; 1 = post-BTX treatment.

Figure 3 shows LHQ score of all patients at baseline and post-BTX treatment. The majority of patients showed an improvement in LHQ following BTX treatment. Linear mixed model analysis was calculated with treatment (“time”) and gender being the fixed factors and patients as random factors. There was a significant effect of the treatment on LHQ outcome (F(1, 25) = 12.335, *p* = 0.002). There was no significant effect of gender (*p* = 0.265) and no significant interaction effect between ’time’ and gender (*p* = 0.078), indicating treatment effects were independent of gender. Parameter estimate showed that regression coefficient (b) for LHQ scores was statistically significant (b = −2.633, t(25.0) = −3.423, *p* = 0.002). After BTX treatment, mean LHQ score increased by 2.6 (95% CI = 1.1–4.2, Sidak-adjusted *p* = 0.002). 

#### 3.2.2. CSI

**Figure 4:**
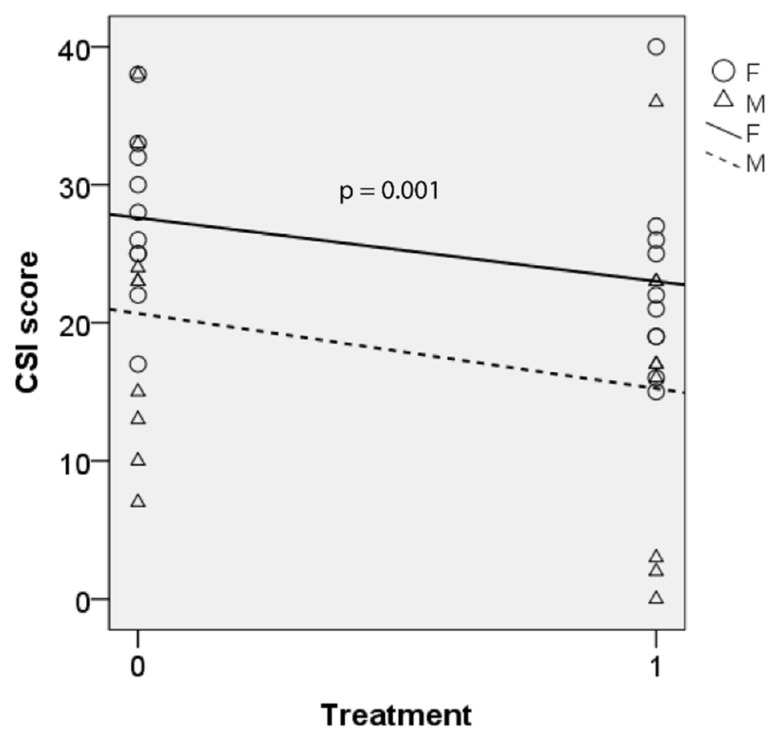
CSI scores before and after BTX therapy with linear trend lines for male (M) and female (F). Lower score indicates better outcome. 0 = baseline; 1 = post-BTX treatment.

Figure 4 shows CSI scores for both genders at baseline and after BTX. There were significant fixed effects of treatment on CSI scores (F(1, 18.998) = 15.068, *p* = 0.001) and no significant interaction between treatment and gender (*p* = 0.748). Parameter estimate showed that CSI score decreased significantly after injection (b = 5.444, t(18.998) = 2.900, *p* = 0.009). Pairwise comparison showed that CSI score decreased by 5.0 after treatment (95% CI = 2.3–7.7, Sidak-adjusted *p* = 0.001).

#### 3.2.3. RSI

**Figure 5:**
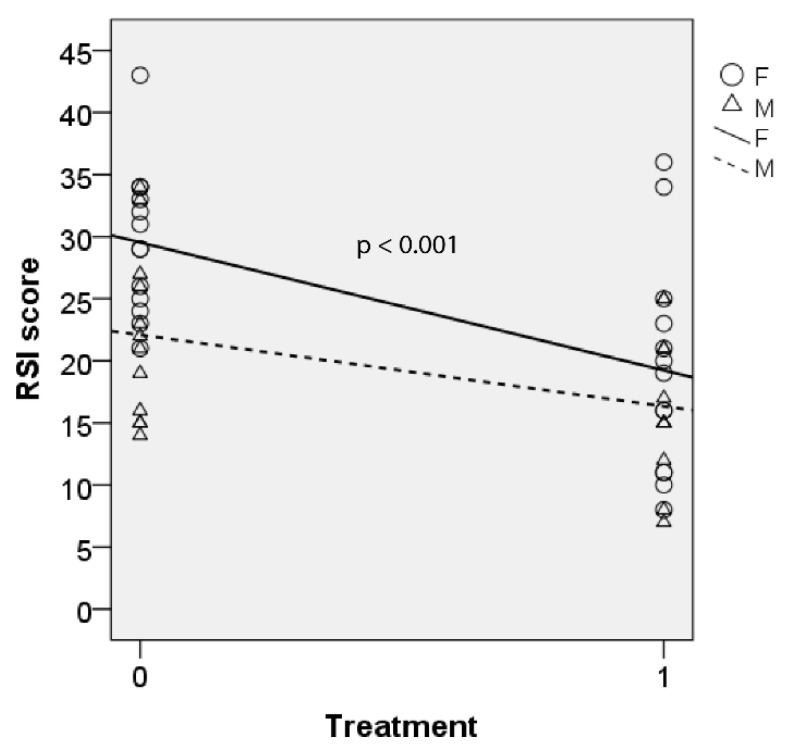
RSI scores before and after BTX therapy with linear trend lines for male (M) and female (F). Lower score indicates better outcome. 0 = baseline; 1 = post-BTX treatment.

Mixed model analysis was calculated for total RSI score which are shown for both males and females in Figure 5. There was significant fixed effect of treatment on total RSI score (F(1, 25.001) = 19.766, *p* < 0.001). There was no significant interaction between treatment and gender (*p* = 0.219). The decrease in RSI score after BTX injection was significant (b = 5.75, t (25.001) = 2.208, *p* = 0.037). Data from both genders showed that the mean RSI scores decreased by 8.0 after BTX injection (95% CI = 4.3–11.8, Sidak-adjusted *p* < 0.001).

Sub-score analysis of the RSI data was also performed using paired *t* test comparing scores of each of the RSI items between pre- and post-BTX. Results of comparisons are presented in Table 8, which showed significant differences with large effect sizes for sensory items related to cough and “breathing difficulties or choking episodes”.

#### 3.2.4. VHI-10

There was no significant fixed effect of treatment on this outcome measure (*p* = 0.734) and there was also no significant interaction between treatment and gender (*p* = 0.196). Pairwise comparison showed that VHI-10 score dropped by 0.7 after BTX (95% CI = −3.6–5.1, Sidak-adjusted *p* = 0.734).

### 3.3. Effect Sizes of the Treatment

Table 9 shows mean differences, *p* value of the paired *t* test and Cohen’s d for all outcome measures. This table shows that the treatment effect was large for the LHQ and RSI outcomes and medium for the CSI.

### 3.4. Prediction of SLN Block Response on BTX Improvement

Short-term response to SLN block was evaluated using a 10-point Likert scale based upon the patient’s specific presenting symptoms. Table 10 presents the number of patients who showed overall improvement after BTX injection versus those who responded to the SLN block. Responses to SLN block was significantly associated with improvement in LHQ scores (χ^2^ (1) = 6.618, *p* = 0.01).

### 3.5. Adverse Effects of BTX Treatment

Ten of the fourteen subjects experienced adverse effects of the BTX treatment. Dysphonia was the most common with weakness, breathiness or reduced volume and projection of the voice. These symptoms were mild and self-limiting, lasting for 2–3 weeks on average. There was no change in VHI-10 at reassessment. One person experienced mild dysphagia and a slower swallow mechanism which also resolved within three weeks.

### 3.6. Repeat Treatments

Six patients presented for repeat treatment. Two patients had a single repeat treatment at three months and five months respectively. One patient had a further two treatments at six and 9 months after the initial. One patient had a total of three treatments at approximately 3-month intervals. Two patients continue to present for repeat treatment with good effect at 3–6 monthly intervals.

## 4. Discussion 

### 4.1. Clinical Presentation of Patients with LSD

Several disorders triggered by one or more sensory stimuli and manifested by hyperkinetic laryngeal dysfunction such as MTD, PVFM, globus and chronic cough have been grouped under “irritable larynx syndrome” [7]. However, the exact role of the dysfunctional sensory pathway in those conditions has not been confirmed by experimental evidence. Unlike motor function which can be examined using electromyography, there is currently no equivalent objective test for sensory function. This has made it challenging to define, explain and evaluate syndromes involving laryngeal hypersensitivity such as LSD. Explanations for these syndromes have been proposed using neuroplastic [7] or neuropathic models [4,76,77]. Examining sensory symptoms of patients with laryngeal sensitivity is therefore necessary to provide the main clinical clusters that may be useful for diagnosis and treatment follow-up.

Symptoms of LSD have been linked to several umbrella conditions in laryngeal hypersensitivity. Vertigan et al. [3] maintained that laryngeal hypersensitivity existed in the context of CRC, PVFM, MTD and globus. They found that laryngeal hypersensitivity was characterized by significantly higher symptom scores than controls in the breathing, cough, swallowing and phonation domains. They also found that within each clinical group of CRC, PVFM and MTD, the scores for the dominant domain were the highest, e.g., the CRC group had the highest cough score and PVFM had the highest breathing scores. Laryngeal paresthesia scores were significantly higher in these groups compared with controls and there were no significant differences in this score across the groups. Laryngeal sensory dysfunction was therefore investigated in the general pivotal syndromes related to phonation, cough, respiration and swallowing rather than in specific throat sensory profiles. However, they did not specifically describe sensory profiles in relevant PROM scales such as LHQ and RSI.

From case history data, the primary presenting symptoms in this cohort of patients were an abnormal throat sensation and CRC. Other symptoms observed with a lower frequency included choking sensation, voice problems, laryngeal dyspnea and problems with swallowing. PROM data were within the pathological ranges for LHQ, CSI, RSI and VHI-10 (Table 6). Videostrobolaryngoscopy was used to exclude other gross laryngeal pathology but was also useful in identifying signs of laryngeal motor impairment associated with sensory dysfunction in patients with vagal neuropathy [8]. Decreased gross vocal fold movement (3/14), abduction lag (6/14) and unequal vocal fold vertical height (10/14) were the main findings in these patients and gave some indication of laterality of peripheral neuropathy.

When examining potential preceding factors associated with onset, several patterns appear evident. Three of the fourteen people reported preceding URTI which has previously been suggested as a cause of vagal neuropathy [8,20]. Three of the fourteen reported preceding occupational inhalational exposure, a recognized trigger factor of irritable larynx syndrome [78]. Trauma to laryngeal nerves is another recognized cause of neuropathic symptoms [29] and was reported in 2/14 people (one iatrogenic during thyroid surgery and one due to external trauma), both of which exhibited motor signs of weakness on videostrobolaryngoscopy. Two of the fourteen reported preceding intubation, the relevance of this is unclear but local irritation of the larynx is one potential mechanism by which sensitization can take place. Four of the fourteen patients could not recall any preceding event.

Ten of the fourteen patients had a favorable response to a trial SLN block, supporting a diagnosis of sensory neuropathy. When considering a diagnosis of LSD, the majority of the following components should be present: ATS or CRC that has failed conventional medical/behavioral therapy; symptoms easily triggered by sensory stimuli; abnormal patient reported outcome measures of laryngeal sensory function (e.g., LHQ +/− RSI); signs of motor asymmetry on laryngeal stroboscopy; favorable response to a trial SLN block.

### 4.2. Treatment Effects of BTX on LSD

The present study is the first to describe the use and investigate the efficacy of supraglottic botulinum toxin type A injection for symptoms associated with Laryngeal Sensory Dysfunction. We postulated that BTX may affect the sensory afferent loop of the cough reflex via multiple mechanisms using a sensory neuropathic model [50,51]. The internal branch of the SLN is the primary laryngeal sensory afferent nerve contributing to a number of important reflexes including cough, swallow, respiration and laryngospasm [16]. It was thus hypothesized that targeting the peripheral sensory receptors in the distribution of this nerve would be a more effective and logical approach than targeting the intrinsic laryngeal musculature (previously described for the treatment of CRC [49,50,51,52]). Our hypothesis and treatment approach appears to be supported by the findings of this study. 

There were statistically significant improvements in the primary patient reported outcome measures of LHQ (improving by 2.6 post-BTX) and CSI (improving by 5.0). The findings suggest a therapeutic effect of supraglottic BTX in the treatment of laryngeal sensory dysfunction. While not mechanistic proof, these findings are in support of the previously discussed peripheral and central sensitization model, and support the use of BTX in the treatment of neuropathic sensory dysfunction.

The findings relating to RSI score are noteworthy. Baseline RSI scores were within the abnormal range [63], despite ongoing medical and behavioral management of laryngopharyngeal reflux at the time of the BTX injection. Sub-item analysis (Table 8) showed significant improvement in the items relating to abnormal sensation; “excess throat mucous or post-nasal drip” and “sensation of something sticking in your throat or a lump in the throat” which are symptoms common to LSD. Improvement was also seen in the sub items relating to cough and breathing difficulties/choking episodes which are potential motor manifestations of laryngeal sensory dysfunction. These findings support the multi-faceted nature of LSD. 

Despite RSI being developed as a tool for LPR symptoms [63], there is a lack of agreement between its score and laryngopharyngeal pH monitoring [79]. The findings of this study support the fact that symptoms reflected in the RSI are not always associated with LPR [80] and may be related to other etiologies including LSD. In light of this, the mechanism of action of BTX on ATS which resulted in improvement in RSI can be interpreted based upon findings from previous research on neuropathic pain involving peripheral nerve injury.

We found that CSI scores decreased significantly after supraglottic BTX injection, supporting its role as a potential treatment for CRC. The therapeutic effects of BTX on cough are thought to stem from its action upon the sensory pathways in modulating the cough and laryngeal adductor reflexes [50,51,52]. It is also possible that diffusion from the injection site into the intrinsic laryngeal adductor muscles may have occurred, producing the effects which have been reported and explained in some previous studies [50,51]; however, we would have expected an associated decrease in voice if this was the primary mechanism of action

In this study, VHI-10 scores did not change significantly despite the common reports of voice change after BTX treatment. This is in line with the mild and temporary nature of dysphonia after laryngeal BTX injections reported elsewhere in the literature [54,68].

### 4.3. The Role of SLN Nerve Block as Predictor of LSD, CRC and Efficacy of BTX

Recent work has explored SLN block as an office-based treatment for chronic refractory cough with a suspected neuropathic cause. In 2018, Simpson [62] reported improvement in cough severity index scores in a cohort of 23 patients where superior laryngeal nerve block was performed using local anesthesia with steroid. In total, 44% of patients had lasting improvement after one treatment but the mechanism of this extended effect remains unclear. Bupivicaine, considered to be the longest lasting local anesthetic, has an analgesic duration of action of only 4–8 h [81]. The addition of steroid to the local anesthetic could theoretically address any inflammation of the superior laryngeal nerve if it happens to be delivered to the site of the nerve inflammation. Twenty eight of the thirty patients treated by Dhillon reported at least a 50% reduction in symptoms along with significant improvement in CSI scores (the only outcome measure employed in this study) after a minimum of three injections [82,83]. Bradley et al. [84] described surgical section of the SLN as a viable option for treatment of selected patients with refractory neuropathic cough. They also however recognized dysphagia and aspiration as potential complications of this treatment.

In our practice we find SLN block a useful tool to assist with diagnosis of LSD and help guide treatment. Patients with laryngeal sensory symptoms persisting despite medical management of laryngeal irritants such as postnasal drip and laryngopharyngeal reflux are offered a trial unilateral SLN block based upon laterality of symptoms and any laryngeal stroboscopic findings that may suggest superior laryngeal nerve paresis. If there is no improvement in symptoms at 20 min compared with baseline, SLN block is offered on the contralateral side. Where symptom improvement is reported, this suggests that the anesthetized nerve or its peripheral receptors and nerve endings play a significant part in the patient’s presentation, supporting a neuropathic diagnosis and offering a potential target for treatment. It is our experience that symptomatic improvement of LSD after SLN block is short term with most patients reporting a duration of effect in the order of hours rather than days before symptoms return.

In the present study, short term response to SLN block was a significant predictor of longer-term response to supraglottic botulinum toxin. Where laryngeal symptoms do not improve with SLN block, a diagnosis of sensory neuropathy is still possible but is likely to involve other sensory branches of the larynx such as the recurrent laryngeal nerve or may be referred from other sites of a neuropathic process in the vagal pathways.

### 4.4. Limitations of This Study

This was a retrospective study; however, we had a high level of data completeness with no patient loss to follow up. When performing supraglottic BTX treatment, it is our experience that the procedure is tolerated much better by the patient with the assistance of laryngeal anesthesia. We used SLN block at the time of BTX treatment for this purpose. Theoretically, some of the treatment effect may be related to the SLN block; however, all patients had reported only short-term response to prior SLN block performed as an independent procedure as part of workup for LSD and a much longer effect of treatment with concurrent BTX treatment. Finally, due to the retrospective nature of this study we were unable to include a separate control group. Future prospective studies investigating this novel treatment for LSD using a control treatment group (perhaps SLN block with BTX vs. SLN block alone) are indicated based on the promising results of the current study.

### 4.5. Recommendations for Assessment and Treatment of CRC

**Figure 6:**
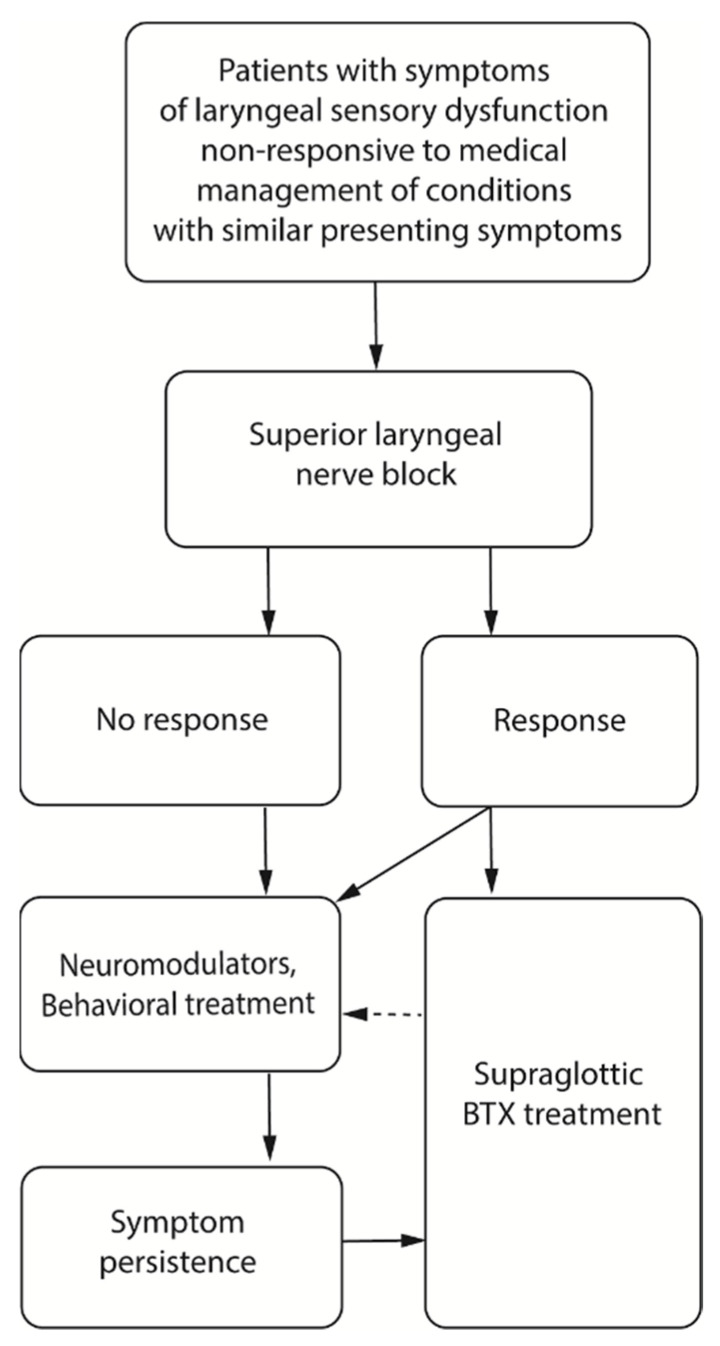
Recommendations of treatment plans for patients with LSD.

This study identified a sub-group of patients presenting with various symptoms within the LSD syndrome and provided preliminary data on the therapeutic effects of BTX administered into a novel supraglottic region of the larynx. This method of BTX administration can be safely performed as an office-based procedure that does not require complicated equipment and concurrent invasive procedures such as laryngeal electromyography. The recommended treatment planning for these patients can be summarized in a flowchart in Figure 6. Patients who present with LSD symptoms are offered superior laryngeal nerve block. If the symptoms improve, supraglottic BTX treatment is indicated. If LSD symptoms do not change after the block, patients will undergo alternative treatments such as medical treatment, neuromodulators, and speech pathology treatment. Those who do not respond to these alternative treatments can be indicated supraglottic BTX as a salvage treatment and they can revert to medical treatment and speech pathology treatment. It is important to mention that clinical trial designs are now required to validate the findings. 

## 5. Conclusions

This study provided further evidence for defining, describing, and diagnosing a subgroup of patients presenting with various laryngeal symptoms related to altered laryngeal sensation. The major presenting symptoms for these patients were abnormal throat sensation and chronic cough. Diagnostic criteria for these patients should be based upon the onset and history of the sensory symptoms, resistance to medical and behavioral treatment, abnormal scores in PROMs evaluating abnormal laryngeal sensation including the LHQ and RSI, laryngeal videostroboscopy findings and responses to SLN block.

Symptomatic immediate response to SLN block supports the diagnosis of LSD affecting the supraglottic laryngeal afferent pathways. It was also a useful predictor of which patients were likely to respond to subsequent treatment with supraglottic BTX injection where the response to SLN block is short-lived.

Supraglottic BTX administration is a safe office-based procedure that effectively reduced sensory symptoms in a cohort of patients with various clinical presentations related to laryngeal sensory dysfunction. This treatment may be considered after the patient fails behavioral intervention and standard medical management for any related co-morbidities such as asthma, laryngopharyngeal reflux or sinonasal conditions including control of potential trigger factors. It can be used as an adjunct to neural modulators or as a standalone treatment to address neuropathic laryngeal symptoms related to LSD including reducing hypersensitivity of the laryngeal afferent pathways and protective reflexes manifesting as chronic refractory cough and throat clearing and reducing sensory symptoms of laryngeal paresthesia presenting as abnormal throat sensation.

## Figures and Tables

**Table 1:** Equivalent laryngeal features of neuropathic pain.

Features of Neuropathic Pain	Explanation	Laryngeal Equivalents
Paresthesia	Abnormal sensation in the absence of a stimulus	Abnormal throat sensation—tickle, lump, globus pharyngeus
Hyperalgesia	Pain triggered at an abnormally low level by a noxious or painful stimulus	Hypertussia—cough triggered at an abnormally low threshold by a recognized cough stimulus
Allodynia	pain triggered by a non-noxious stimulus	Allotussia—cough triggered by non-cough stimuli, e.g., talking (mechanical) or cold air (thermal)

**Table 2:** Characteristics of the treatment cohort. NM, neuromodulator; SLN, superior laryngeal nerve; Gaba, gabapentin; PR, partial response; Ami, amitriptyline; NR, no response; URTI, upper respiratory tract infection; NS, nonsmoker, FS, former smoker.

Patient ID	Potential Preceding Factors Reported	Duration of Symptoms at Presentation (months)	Age, Gender	Smoking	Resp. Disease	Neuromodulator Treatment History
1	URTI	12	44, F	FS	Nil	Past Gaba—PR Ami current—PR
2	Occupational inhalational exposure	1	42, F	FS	Nil	Past Gaba—PR
3	Intubation for hernia surgery	24	58, F	FS	Asthma, OSAS	Past Gaba—side effects
4	nil	5	76, M	FS	Asthma	Nil
5	Occupational inhalational exposure	14	56, M	FS	Nil	Past Gaba—side effects
6	Laryngeal trauma involving superior laryngeal nerve	11	48, M	FS	Nil	Ami current—PR, Past Gaba—side effects
7	URTI	120	32, M	FS	Nil	Declined
8	URTI	120	68, M	FS	Nil	Past Gaba—NR Ami current—PR
9	Occupational inhalational exposure	15	55, M	NS	Nil	Past Ami—side effects, Gaba current—PR
10	Intubation for cosmetic surgery	180	75, F	NS	Nil	Ami current—PR
11	nil	36	56, M	NS	Nil	Ami—side effects Gaba current—PR
12	nil	360	60, M	NS	Asthma (mild, controlled)	Nil
13	nil	240	44, F	NS	Nil	Past Gaba—NR
14	Thyroid surgery with Vocal fold palsy	7	54, F	NS	Nil	Past Ami—Side effects & NR Past Gaba—side effects & PR

**Table 3:** Intra-rater reliability (exact agreement in second rating/total repeated videos).

Parameters	Rater 1	Rater 2
Vocal fold movement	7/7	7/7
Abduction lag	7/7	6/7
Axis rotation on pitch glide	7/7	7/7
Phase symmetry	7/7	7/7
Amplitude	7/7	7/7

**Table 4:** Inter-rater reliability of strobolaryngoscopy ratings.

Parameters	Exact Agreement/Total Videos
Vocal fold movement	13/14
Abduction lag	8/14
Axis rotation on pitch glide	12/14
Vertical vocal fold mismatch	9/14
Phase symmetry	10/14

**Table 5:** Clinical characteristics. CC, chronic cough; ATS, abnormal throat sensation; LD, laryngeal dyspnea.

Patients	Primary Presenting Symptom/s	Secondary Symptoms
1	CC, ATS	Dysphonia, LD
2	Dysphonia, ATS	LD
3	LD, CC, ATS	Dysphagia
4	LD, choking	Dysphonia
5	ATS, dysphagia	CC, dysphonia
6	ATS, CC	Dysphonia, dysphagia, choking
7	ATS, CC	Choking
8	CC	Choking
9	CC, ATS, LD	Throat pain
10	CC, ATS	Dysphonia
11	CC, ATS	Choking
12	CC, ATS	
13	CC, ATS	
14	ATS, dysphonia	CC, choking

**Table 6:** Descriptive statistics of patient reported outcome measures at baseline.

PROMs	Mean (SD)	95% CI	Abnormal Value
LHQ	12.81 (3.418)	11.16–14.45	<17.1 [55]
CSI	24.32 (8.870)	20.04–28.59	≥3 [60]
RSI	27.37 (6.946)	24.02–30.72	≥13 [63]
VHI 10	18.37 (10.308)	13.40–23.34	≥11 [69]

**Table 7:** Stroboscopy findings in LSD.

Parameters	Ratings	Number
Gross VF movement	Normal	11
	Decreased	2
	Absent	1
Abduction lag	Yes	6
	No	8
Axis Rotation on Pitch Glide	Yes	4
	None	10
VF length	Equal	9
	One VF shorter	5
Vertical Level on Phonation	On plane	4
	One VF lower	10
Phase symmetry	In phase	14
	Out of phase	0
Phase Closure	Normal	11
	Closed phase	3
Amplitude	Normal	14
	Abnormal	0
Periodicity	Regular	13
	Irregular	1
False Vocal Fold Hyperfunction	None	8
	Unilateral	6
Supraglottic lateral constriction	Severe	5
	Moderate	4
	Mild	5
Supraglottic AP constriction	Severe	1
	Moderate	2
	Mild	6
	None	5
Mucosal lesions	Yes	1
	No	13

**Table 8:** Results of paired *t* test and effect size for RSI items (*n* = 25). All items were quoted verbatim from the original RSI scale by Belafsky et al. [63]. Cohen’s d: small = 0.2; medium = 0.5; large = 0.8 [75]. MD, mean difference; (*), significant at *p* < 0.05.

RSI Items (from Reference [63])	MD	t	*p*	Cohen’s D
“Hoarseness or a problem with your voice”	0.7	1.737	0.095	0.483
“Clearing your throat”	0.6	1.995	0.058	0.497
“Excess throat mucous or post-nasal drip”	0.6	2.777	0.010 *	0.388
“Difficulty swallowing food, liquids or pills”	1.0	3.062	0.005 *	0.560
“Coughing after you ate or after lying down”	0.8	3.199	0.004 *	0.942
“Breathing difficulties or choking episodes”	1.6	5.286	0.001 *	1.163
“Troublesome or annoying cough”	1.2	4.243	0.001 *	0.923
“Sensations of something sticking in your throat or a lump in your throat”	1.4	3.395	0.002 *	0.788
“Heartburn, chest pain, indigestion or stomach acid coming up”	0.2	0.451	0.656	0.093

**Table 9:** Mean, mean difference, and effect sizes (Cohen’s d: small = 0.2; medium = 0.5; large = 0.8). MID, minimal clinically important difference; (*), significant at *p* < 0.05.

Measures		Mean (SD)	N	Mean Difference	MID	*p*	d
LHQ	Pre	13.07 (3.288)	25	2.633	1.7	0.003 *	−0.800
	Post	15.70 (3.086)	25				
CSI	Pre	24.32 (8.870)	19	5.000	3 [60]	0.001 *	0.564
	Post	19.32 (10.193)	19				
RSI	Pre	25.96 (7.311)	25	8.120	4	0.001 *	1.111
	Post	17.84 (7.493)	25				
VHI-10	Pre	18.20 (9.916)	25	0.840	6	0.710	0.085
	Post	17.36 (8.850)	25				

**Table 10:** Overall BTX improvement versus outcome of SLN block.

		Overall BTX Improvement		Total	*p*
		No	Yes		
SLN block response	No	4 (16.0%)	1 (4.0%)	5 (20.0%)	0.01
	Yes	4 (16.0%)	16 (64.0%)	20 (80.0%)	
Total		8 (32.0%)	17 (68.0%)	25 (100%)	

## Data Availability

Data supporting reported results is retained by The University of Sydney in de-identified form and is confidential under the conditions of the Human Research Ethics Committee of The University of Sydney approval.
